# Supplementary material for: Feasibility of Patient Reported Outcome Measures in Psychosocial Palliative Care: Observational Cohort Study of Hospice Day Care and Social Support Groups
Source: Int J Environ Res Public Health. 2022 Oct 14;19(20):13258. doi: 10.3390/ijerph192013258 (PMC9603547; doi:10.3390/ijerph192013258)
Supplement: Supplementary file 1 [file ijerph-19-13258-s001.zip › ijerph-1908367-supplementary.pdf]

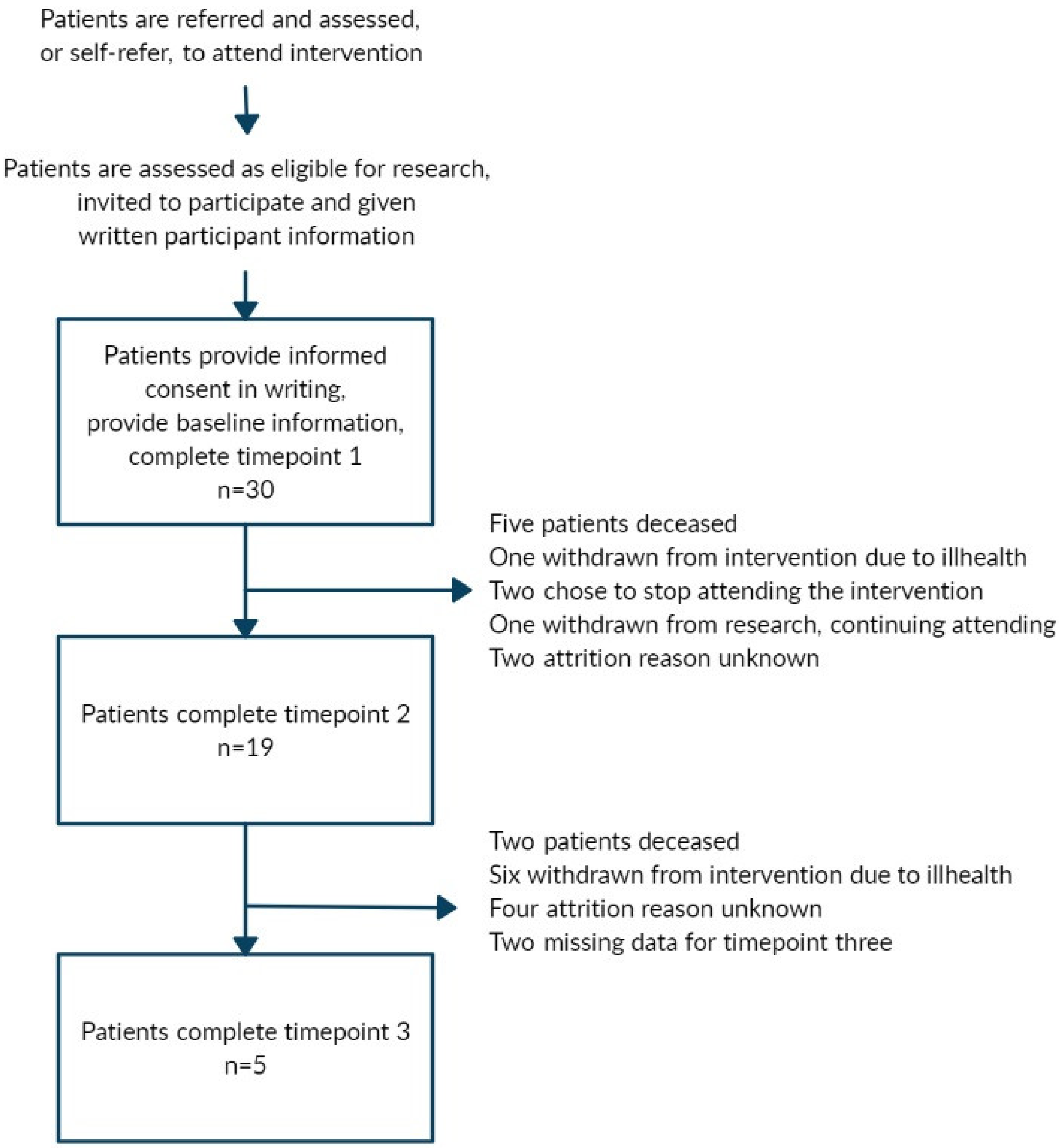

Figure S1: Data collection across all four research locations

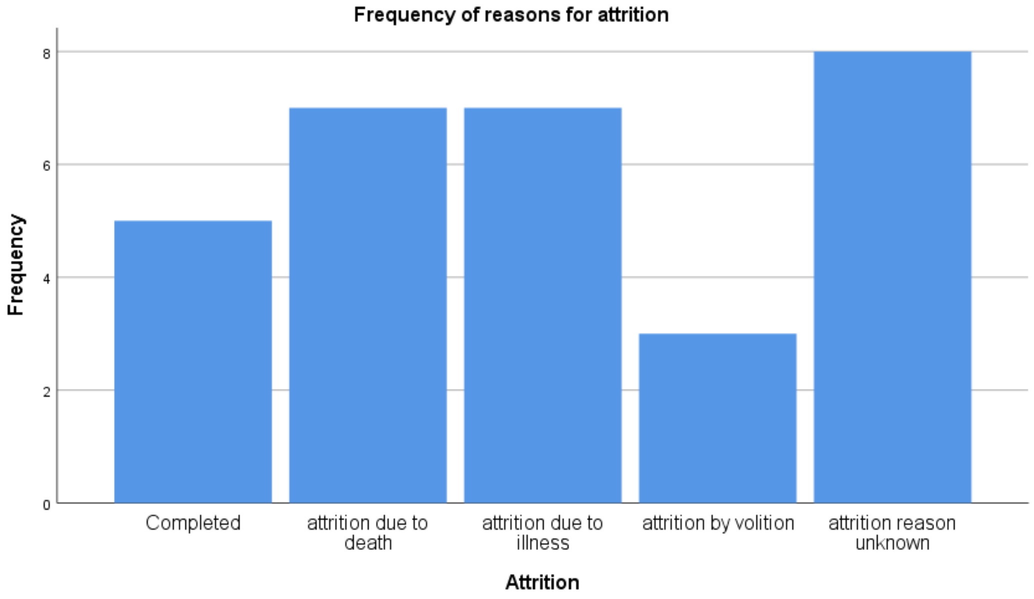

Figure S2: Frequency of reasons for attrition, with those that completed all three timepoints

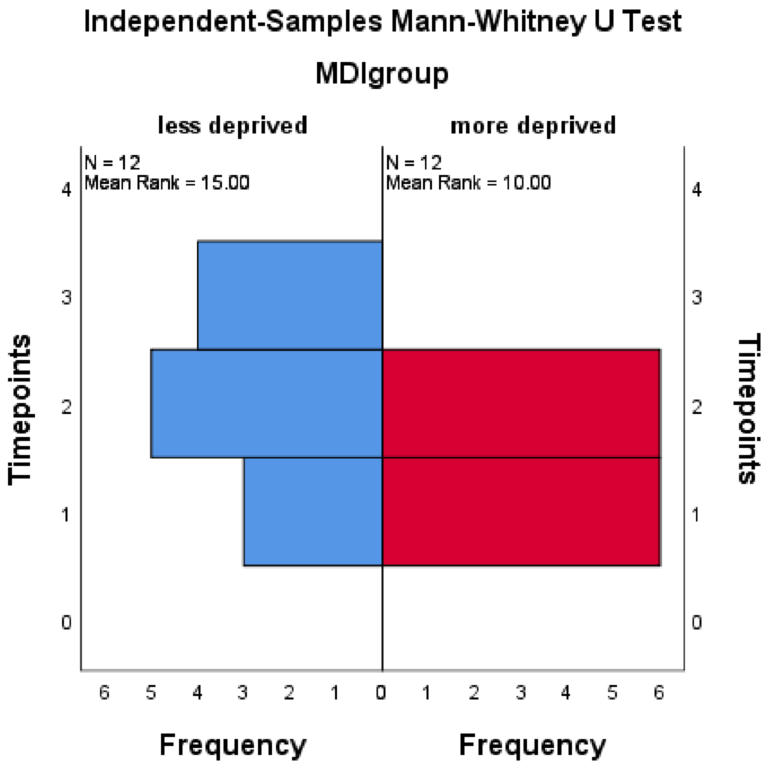

Figure S3: Timepoints collected from patients by deprivation index

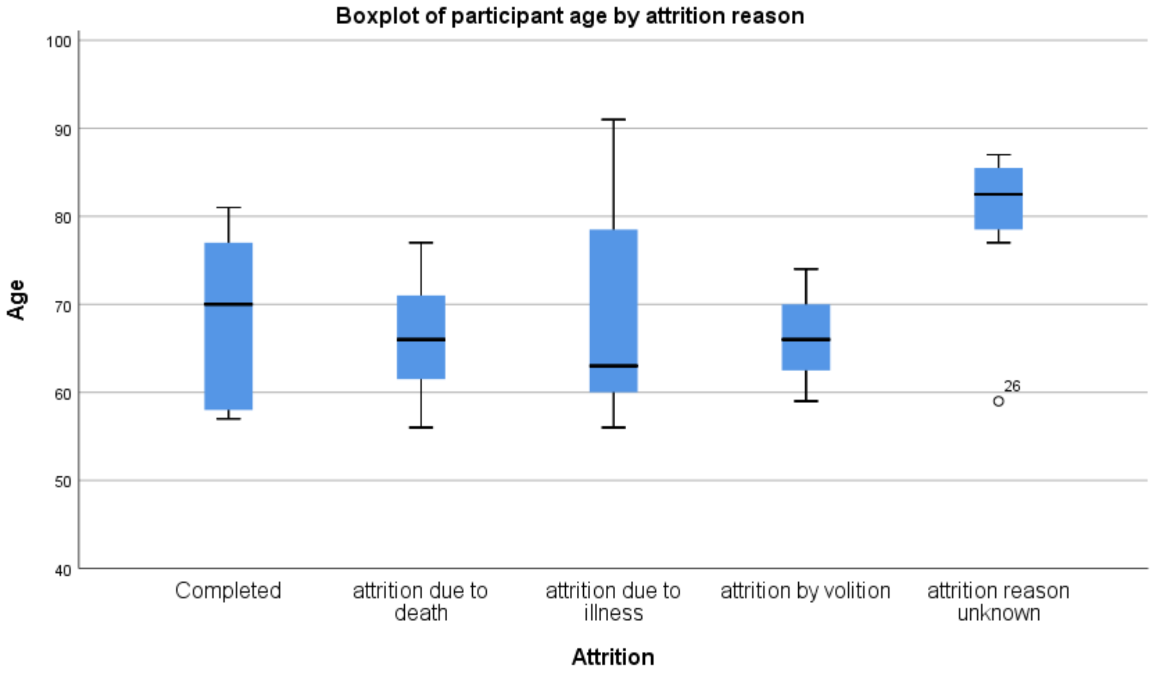

Figure S4: Boxplot showing participant age by attrition reason

Table S1: Results of psychosocial outcome measures for whole sample

	T1 (n=30)	T2 (n=19)	T3 (n=5)
**Perceived social support**			
Mean	80.139	80.158	87.200
Standard error of mean	2.729	4.230	5.536
Min value	50.50	22.00	75.16
Max value	100	100	100
Standard deviation	14.945	18.437	12.380
**Loneliness**			
Mean	5.30	5.11	4.20
Standard error of mean	0.326	0.518	0.374
Min value	3	3	3
Max value	9	9	5
Standard deviation	1.784	2.258	0.837
**Depression**			
Mean	6.82	7.11	5.60
Standard error of mean	0.675	0.791	0.872
Min value	2	2	3
Max value	15	13	8
Standard deviation	3.696	3.446	1.949

Table S2: Descriptive statistics of perceived social support observed over time (all data), figures to 3 decimal places

	T1 (n=30)	T2 (n=19)	T3 (n=5)
**Emotional support**			
Mean	80.191	82.368	87.000
Standard error mean	3.019	4.653	8.116
Standard deviation	16.534	20.284	18.149
**Tangible support**			
Mean	79.667	81.579	81.000
Standard error mean	3.690	4.557	8.426
Standard deviation	20.211	19.864	18.841
**Affectionate support**			
Mean	86.667	85.263	90.664
Standard error mean	3.306	4.771	4.001
Standard deviation	18.110	20.796	8.946
**Social interaction**			
Mean	76.667	76.843	89.332
Standard error mean	3.541	4.738	4.521
Standard deviation	19.395	20.652	10.111
**Overall social support**			
Mean	80.138	80.158	87.200
Standard error mean	2.738	4.230	5.536
Standard deviation	14.946	18.437	12.379

a.

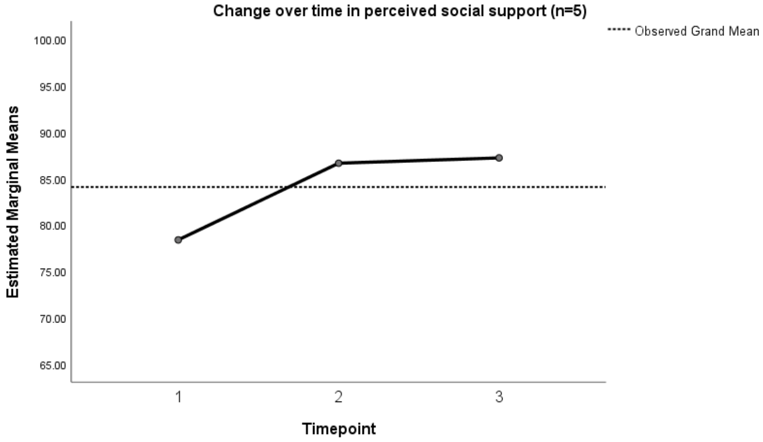

b.

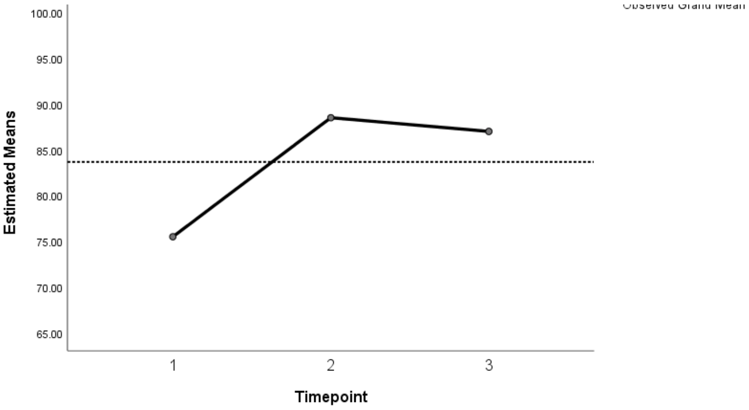

c.

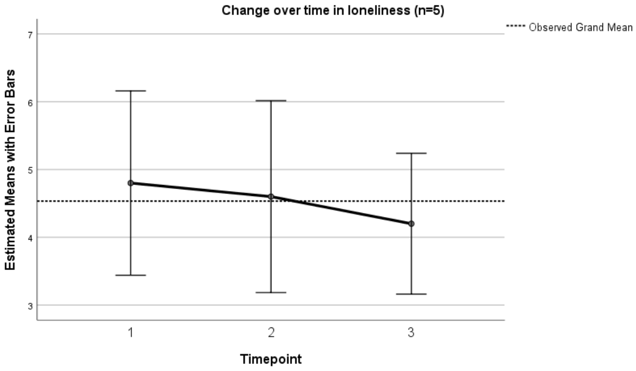

d.

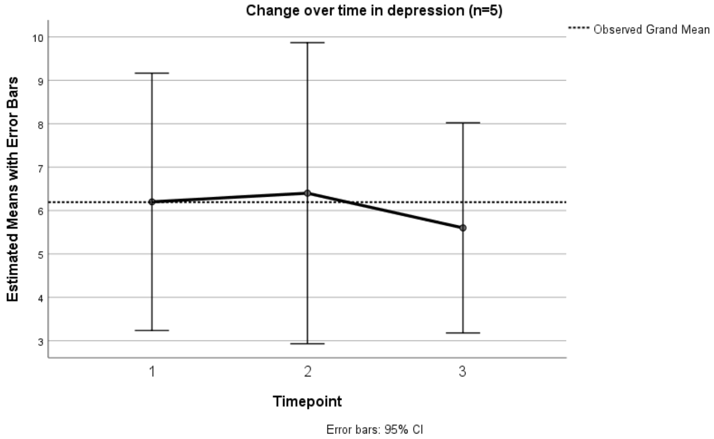

Figure S5: Change between T1 and T3 in (a) Perceived social support; (b) Emotional support; (c) Loneliness; (d) Depression

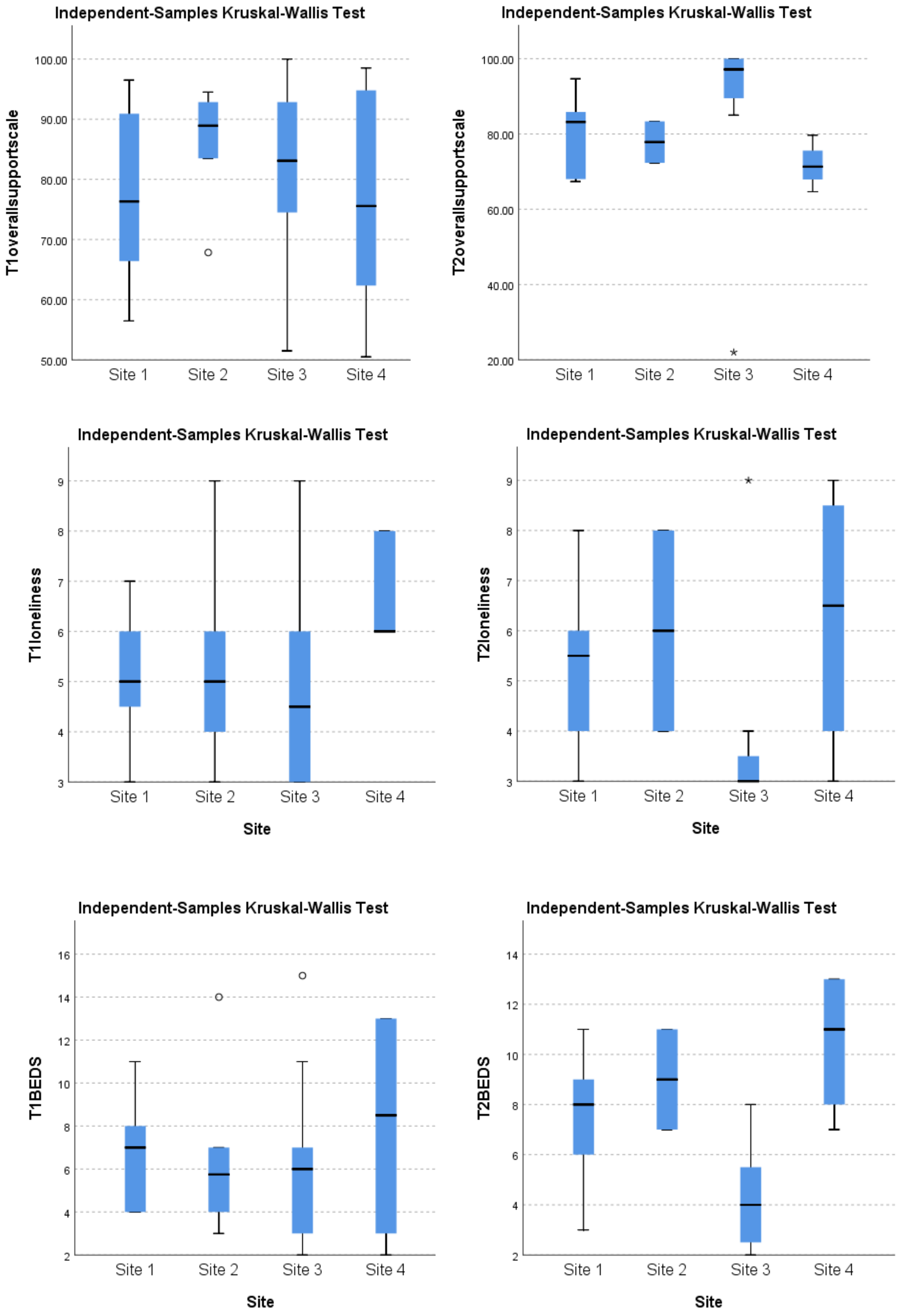

Figure S6: Differences between sites at baseline were non-significant across measures at T1. Differences in depression were significant at T2, depicted in bottom-right panel.

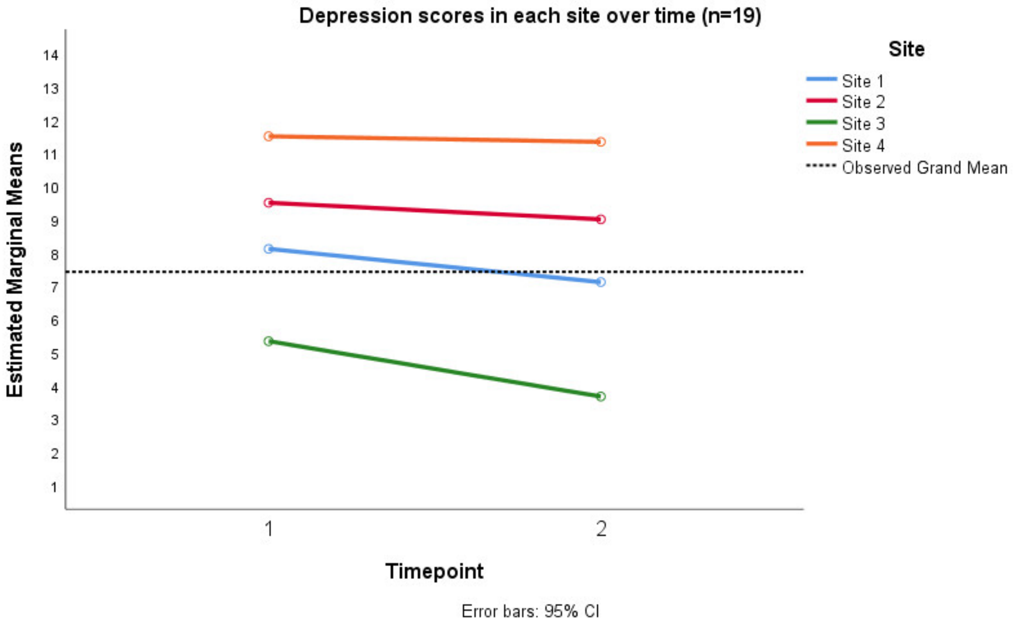

Figure S7: Mean depression score for each site at T1 and T2 (n=19)

a.

b.

c.

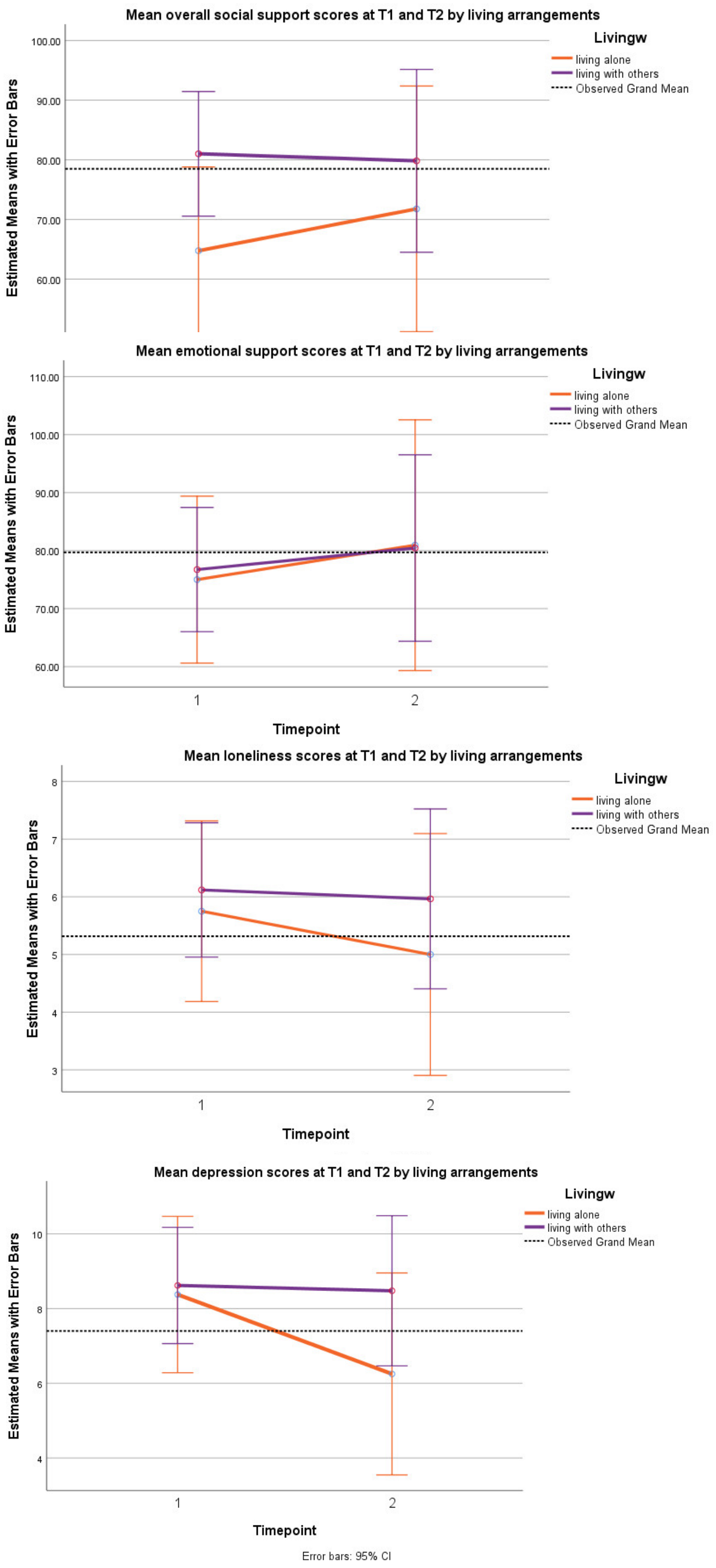

Figure S8: Change over between T1 and T2, (n=19), comparing participants who lived alone and those who lived with others in: (a) Perceived social support; (b) Emotional support; (c) Loneliness; (d) Depression.

a.

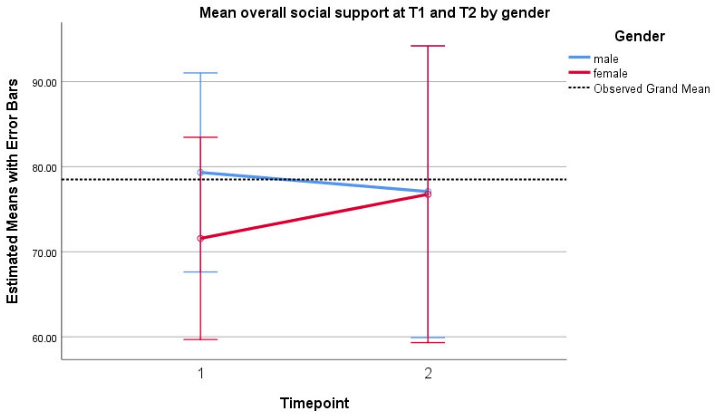

b.

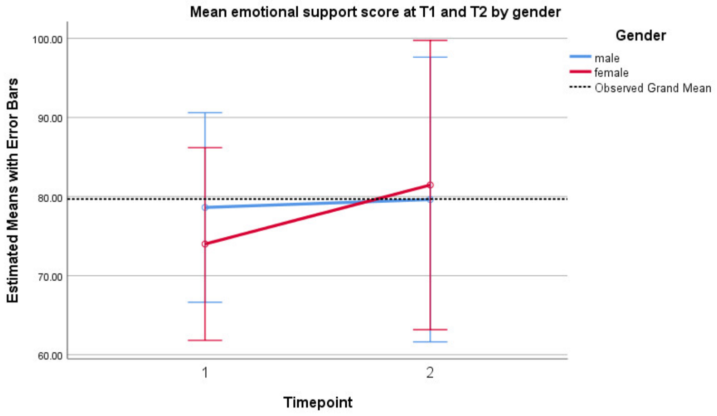

c.

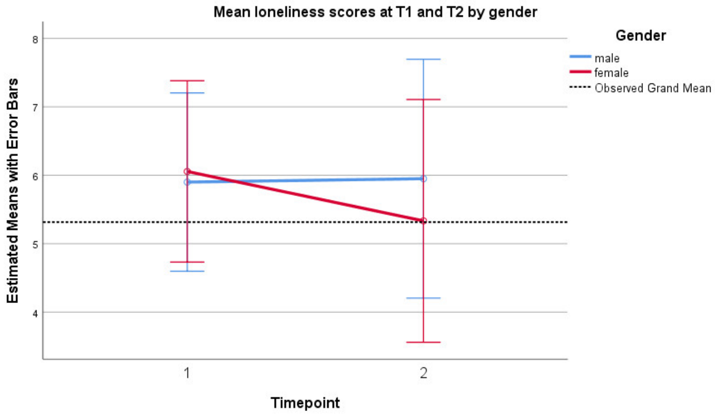

d.

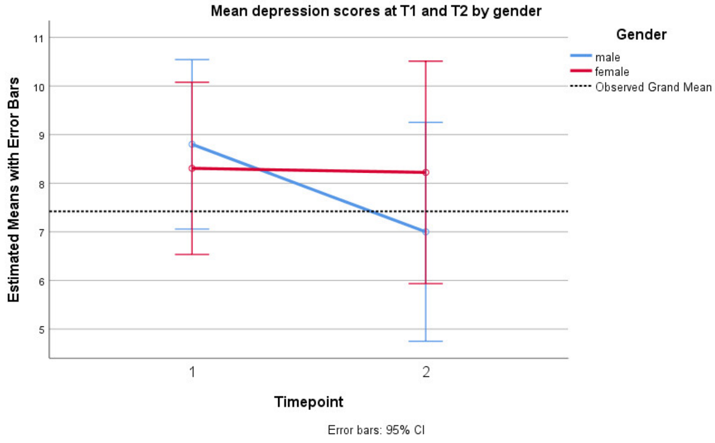

Figure S9: Change over time (n=19), comparing men and women in: (a) Perceived social support; (b) Emotional support; (c) Loneliness; (d) Depression.

Appendix S1: Participant Information Sheet

Academic Palliative and Supportive Care Studies Group,

Institute of Psychology, Health and Society,

Waterhouse Building (Block B),

1-5 Brownlow Street,

University of Liverpool,

Liverpool.

L69 3GL.

PARTICIPANT INFORMATION SHEET

Title of Study: Social Support in Palliative Care

You are being invited to take part in a research study. Before you decide whether or not to participate, it is important that you understand why the research is being undertaken and what it will involve. Please take the time to read the following information and feel free to ask us if you would like more information or if there is anything that you do not understand. You may also wish to discuss this with your family, friends or doctor. We would like to stress that you do not have to accept this invitation and should only take part if you wish to do so. Thank you for reading this information.

What is the purpose of this study?

Social isolation and loneliness is a significant issue in our society. The project aims to explore different forms of social support, focussing on services that offer people living in the community the opportunity to gain social support. In a context of increasing demand and decreasing resources within health and social care, it is important for research to consider how services are provided and how they can show that they are effective.

Why have I been chosen to take part?

We are looking for participants that are about to start or have recently started attending one of the services that we are studying. You have been referred to us by a member of staff at [insert service]. We would like to ask you to complete some written questionnaires over 12 weeks, to give us information about your experiences attending the service.

Do I have to take part?

It is up to you whether you decide to take part, it is entirely voluntary. You can choose to withdraw from the study without being affected in any way. This information sheet is yours to keep.

What will happen if I decide to take part?

If you do decide to take part, you will be asked to sign a consent form. You will be given written questionnaires to complete and you are allowed to ask someone to help you complete the questionnaires. You will be asked to fill in the questionnaires now, in 6 weeks, and again in another 6 weeks. The questionnaires will be returned to the researcher in pre-paid envelopes provided with the questionnaires.

What are the benefits of taking part?

There are no direct benefits to you for taking part in this study. By taking part, you will help us to find out more about what is important and effective about social support. A summary of findings will be available to participants.

What if there is a problem or complaint?

If a problem arises please contact Natasha Bradley of 0796 901 5126 and we will try to resolve the problem. If you remain unhappy please contact the University Research Governance Officer on 0151 794 8290, providing the name of the researcher and the name of this study (given at the top of this letter).

Will the information I give be kept confidential?

The information that you give us will be confidential and stored securely. Every person taking part in the study will be allocated a unique number so that no information that might be used to identify you will be stored. Only the research team will have access to the data that is collected. It is likely that this research will be published in journals and presented at conferences, however all participants will remain anonymous.

What will happen if I decide I do not want to continue taking part?

It is not a problem if at any time you decide you do not want to carry on taking part in the study. Your participation is voluntary and you can withdraw without any consequences to you. You will be asked to contact Natasha Bradley to tell her that you no longer wish to take part.

Who do I contact if I have questions about this study?

Please contact Natasha Bradley on 0796 901 5126 or Natasha.bradley@liverpool.ac.uk.

Appendix S2: Consent Form

Title of Study: **Social Support in Palliative Care**

Researcher(s): **Natasha Bradley**
Supervisor: Professor Mari Lloyd-Williams

Please initial box

1. I confirm that I have read and have understood the information sheet for the above study. I have had the opportunity to consider the information, ask questions and have had these answered satisfactorily. ☐

2. I understand that my participation is voluntary and that I am free to withdraw without giving any reason, without my rights being affected. In addition, should I not wish to answer any particular question or questions, I am free to decline. ☐

3. I understand that, under the Data Protection Act, I can at any time ask for access to the information I provide and I can also request the destruction of that information if I wish. ☐

4. I understand that confidentiality and anonymity will be maintained and it will not be possible to identify me in any publications. ☐

5. I understand that any concerns about me or someone else being in potential danger will be addressed in line with University of Liverpool Guidelines. ☐

6. I understand and agree that my participation will involve completing questions at three time points, I consent to my answers being stored securely for data analysis. ☐

7. I agree to take part in the above study. ☐

-

______________________	________	______________
Name of Participant	Date	Signature
______________________	________	______________

Name of Person taking consent Date Signature

Appendix S3: Participant Details

Name: ____

Telephone: ____

Home postcode: ____

Please describe your current living arrangements:

☐ I live alone

☐ I live with other people: _______adults and _____ children

Please give your age: __________ DOB: ________________

Please indicate your gender:

M[illegible]☐ Fe☐[illegible]e Pr☐[illegible]not to say

Please indicate your ethnicity (please tick one box):

☐ White ☐ Asian

☐ Mixed ethnicity ☐ Black

☐ Any other ethnic background ☐ Prefer not to say

What country were you born in? ______________________________________

Do you have any long-term health conditions (*e.g. arthritis, cancer (please specify), dementia, diabetes, epilepsy, hearing loss, heart disease, osteoporosis, motor neurone disease, Parkinson's, renal disease, respiratory disease/COPD, stroke*)?
Please list in the space below:

____

____

____

____

Appendix S4: Participant Question Pack

**Participant ID:**	**Date today:**

W[illegible]-2

W[illegible]-8

W[illegible]2-14

This pack contains 36 questions in two sections. Each section opens with a brief instruction. Please choose one of the multiple choice answers for each question.

It is important that an answer is given for each question. You can choose not to answer any of the questions, in this case please tick ‘declined to answer’.

Primary researcher: Natasha Bradley
07969015126
Natasha.bradley@liverpool.ac.uk

Part 1: Social support

People sometimes look to others for companionship, assistance, and other types of support. How often is each of the following kinds of support available to you if you need it?

1. **Someone you can count on to listen to you when you need to talk**

None of the time ☐ A little of the time ☐ Some of the time ☐ Most of the time ☐ All of the t ☐

Declined to answer ☐

2. **Someone to give you information to help you understand a situation**

None of the time ☐ A little of the time ☐ Some of the time ☐ Most of the time ☐ All of the t ☐

Declined to answer ☐

3. **Someone to give you good advice about a crisis**

None of the time ☐ A little of the time ☐ Some of the time ☐ Most of the time ☐ All of the ti ☐

Declined to answer ☐

4. **Someone to confide in or talk to about yourself or your problems**

None of the time ☐ A little of the time ☐ Some of the time ☐ Most of the time ☐ All of the ti ☐

Declined to answer ☐

5. **Someone whose advice you really want**

None of the time ☐ A little of the time ☐ Some of the time ☐ Most of the time ☐ All of the t ☐

Declined to answer ☐

6. **Someone to share your most private worries and fears with**

None of the time ☐ A little of the time ☐ Some of the time ☐ Most of the time ☐ All of the t ☐

Declined to answer ☐

7. **Someone to turn to for suggestions about how to deal with a personal problem**

None of the time ☐ A little of the time ☐ Some of the time ☐ Most of the time ☐ All of the t ☐

Declined to answer ☐

8. Someone who understands your problems

None of the time ☐ A little of the time ☐ Some of the time ☐ Most of the time ☐ All of the t ☐

Declined to answer ☐

9. Someone to help you if you were confined to bed

None of the time ☐ A little of the time ☐ Some of the time ☐ Most of the time ☐ All of the t ☐

Declined to answer ☐

10. Someone to take you to the doctor if you needed it

None of the time ☐ A little of the time ☐ Some of the time ☐ Most of the time ☐ All of the t ☐

Declined to answer ☐

11. Someone to prepare your meals if you were unable to do it yourself

None of the time ☐ A little of the time ☐ Some of the time ☐ Most of the time ☐ All of the t ☐

Declined to answer ☐

12. Someone to help with daily chores if you were sick

None of the time ☐ A little of the time ☐ Some of the time ☐ Most of the time ☐ All of the t ☐

Declined to answer ☐

13. Someone who shows you love and affection

None of the time ☐ A little of the time ☐ Some of the time ☐ Most of the time ☐ All of the t ☐

Declined to answer ☐

14. Someone to love and make you feel wanted

None of the time ☐ A little of the time ☐ Some of the time ☐ Most of the time ☐ All of the t ☐

Declined to answer ☐

15. Someone who hugs you

None of the time A little of the time Some of the time Most of the time All of the t

Declined to answer

16. Someone to have a good time with

None of the time A little of the time Some of the time Most of the time All of the t

Declined to answer

17. Someone to get together with for relaxation

None of the time A little of the time Some of the time Most of the time All of the t

Declined to answer

18. Someone to do something enjoyable with

None of the time A little of the time Some of the time Most of the time All of the t

Declined to answer

19. Someone to do things with to help you get your mind off things

None of the time A little of the time Some of the time Most of the time All of the t

Declined to answer

20. How often do you feel that you lack companionship?

Hardly ever Some of the time Often

Declined to answer

21. How often do you feel left out?

Hardly ever Some of the time Often

Declined to answer

22. How often do you feel isolated from others?

Hardly ever Some of the time Often

Declined to answer

Part 2: Wellbeing and health

For these questions, please choose the answer which comes closest to how you have felt **in the past 7 days** (not just how you feel today).

23. I have blamed myself unnecessarily when things have gone wrong

- Yes, most of the time ☐
- Yes, some of the time ☐
- Not very often ☐
- No, never ☐

Declined to answer ☐

24. I have been so unhappy that I have had difficulty sleeping

- Yes, most of the time ☐
- Yes, quite often ☐
- Not very often ☐
- No, not at all ☐

Declined to answer ☐

25. I get a sort of frightened feeling as if something awful is about to happen

- Yes, definitely and quite badly ☐
- Yes, but not too badly ☐
- A little, but it doesn't worry me ☐
- Not at all ☐

Declined to answer ☐

26. I have felt sad or miserable

- Yes, most of the time ☐
- Yes, quite often ☐
- Not very often ☐
- No, not at all ☐

Declined to answer ☐

27. Things have been getting on top of me

- Most of the time and I haven't been able to cope at all ☐
- Yes, sometimes I haven't been coping as well as usual ☐
- No, most of the time I have coped quite well ☐
- No, I have been coping as well as ever ☐

Declined to answer ☐

28. The thought of harming myself has occurred to me

- Yes, quite often ☐
- Sometimes ☐
- Hardly ever ☐
- Never ☐

Declined to answer ☐

For these questions, please choose one answer that best describes your health **today.**

29. Mobility – do you have problems in walking about?

- ☐ I have no problems in walking about
- ☐ I have slight problems in walking about
- ☐ I have moderate problems in walking about
- ☐ I have severe problems in walking about
- ☐ I am unable to walk about

Declined to answer ☐

30. Self-care – do you have problems washing or dressing yourself?

- ☐ I have no problems washing or dressing myself
- ☐ I have slight problems washing or dressing myself
- ☐ I have moderate problems washing or dressing myself
- ☐ I have severe problems washing or dressing myself
- ☐ I am unable to wash or dress myself

Declined to answer ☐

31. Usual activities (e.g. shopping, housework, family or leisure activities)

- ☐ I have no problems doing my usual activities
- ☐ I have slight problems doing my usual activities
- ☐ I have moderate problems doing my usual activities
- ☐ I have severe problems doing my usual activities
- ☐ I am unable to do my usual activities

Declined to answer ☐

32. Pain/discomfort – how is your pain or discomfort today?

- ☐ I have no pain or discomfort
- ☐ I have slight pain or discomfort
- ☐ I have moderate pain or discomfort
- ☐ I have severe pain or discomfort
- ☐ I have extreme pain or discomfort

Declined to answer ☐

33. Anxiety/depression – do you feel anxious or depressed today?

- ☐ I am not anxious or depressed
- ☐ I am slightly anxious or depressed
- ☐ I am moderately anxious or depressed
- ☐ I am severely anxious or depressed
- ☐ I am extremely anxious or depressed

Declined to answer ☐

34. In the last 6 weeks, have you visited hospital for A&E (casualty)?

☐ Ye[illegible]ore than once) Total number of visits: __________

☐ Yes (once)

☐ No

Declined to answer ☐

35. In the last 6 weeks, have you stayed in hospital overnight?

☐ Yes Total number of nights: __________

☐ No

Declined to answer ☐

Final Question

- We would like to know how good or bad your health is TODAY.
- This scale is numbered from 0 to 100.
- 100 means the best health you can imagine.
  0 means the worst health you can imagine.
- Mark an X on the scale to indicate how your health is TODAY.
- Now, please write the number you marked on the scale in the box below.

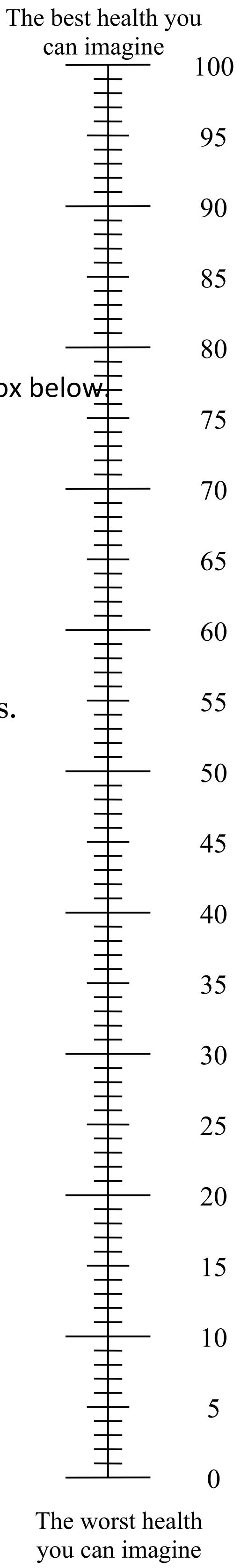

YOUR HEALTH TODAY = ☐

Declined to answer ☐

Thank you for contributing your time in completing these questions.
